# Influence of the drone brood homogenate on the gut integrity and cellular immunity: A pilot study on pigs

**DOI:** 10.17221/98/2025-VETMED

**Published:** 2026-03-30

**Authors:** Viera Karaffova, Dagmar Mudronova, Martin Levkut, Lubica Rajcakova, Erik Hudec, Rudolf Zitnan, Peter Patras

**Affiliations:** ^1^Department of Morphological Disciplines, University of Veterinary Medicine and Pharmacy, Košice, Slovak Republic; ^2^Department of Microbiology and Immunology, University of Veterinary Medicine and Pharmacy, Košice, Slovak Republic; ^3^Research Institute for Animal Production, National Agricultural and Food Centre, Lužianky, Slovak Republic

**Keywords:** Apilarnil, gut integrity, lymphocyte subpopulations, phagocytosis, tight junctions

## Abstract

Drone brood homogenate (DBH), a nutrient-rich bee product, has received limited scientific attention despite its potential immunomodulatory and gut-protective properties. This study evaluated the effects of a dietary DBH supplementation on the intestinal barrier–related gene expression, phagocytic activity, and lymphocyte subpopulations in pigs. Eighteen weaned pigs were assigned to three groups (control, DBH100, DBH200) and fed DBH at 0, 100, or 200 mg/kg feed for 18 days. The gene expression of tight junction markers (occludin, claudin-1) and mucosal integrity–associated proteins (lumican, OLFM4) was assessed in the ileum by qRT-PCR. Phagocyte function and peripheral blood lymphocyte subpopulations were analysed by flow cytometry. DBH200 significantly upregulated the occludin, claudin-1, lumican, and OLFM4 expression, indicating enhanced intestinal barrier support. The phagocytes from both DBH-treated groups exhibited an increased engulfing capacity and an elevated oxidative burst index, though the percentage of active phagocytes was only weakly affected. The DBH supplementation did not alter the total T (CD3^+^) or B (CD21^+^) cells; however, both DBH groups showed a significantly increased CD4^+^ : CD8^+^ lymphocyte ratio, which is consistent with immune stimulation. These findings suggest that DBH may beneficially modulate the gut barrier integrity and selected components of innate and adaptive cellular immunity in pigs.

The overuse of antibiotics in livestock production is well recognised as a major factor contributing to the global development and dissemination of antimicrobial-resistant bacteria. Antibiotics are currently classified as contaminants that pose a significant threat to public health worldwide (Xu et al. 2022). Exploiting the potential of bee products, whether as part of the human food spectrum or as an ingredient in animal feed, could be an alternative in view of the growing resistance to antibiotics and, at the same time, help maintain the integrity of the intestinal barrier.

Bee products have been used in natural medicine for centuries. Most scientific reports focus on the nutritional properties and therapeutic effects of propolis, royal jelly, honey, pollen, and bee venom. Less information is available on the drone brood, which represents another potentially useful bee product (Sawczuk et al. 2019). Drone honeybee larvae originate from unfertilised eggs and develop into male individuals. Their primary biological role is to mate with the queen, thereby ensuring the colony’s continuation. Beyond reproduction, drones contribute little to colony maintenance and mainly consume the food resources gathered by worker bees. In addition, the most important bee parasite – *Varroa destructor* prefers the drone brood for its reproduction (Wantuch and Tarpy 2009). Consequently, beekeepers commonly remove the surplus drone brood from hives (Durazzo et al. 2021). The removed drone brood at 6–7 days of age can subsequently be used as a natural nutritional supplement, also known as Apilarnil. Apilarnil or the drone brood homogenate (DBH) has a rich nutritional profile. It contains 8–15% proteins, comprising up to 20 amino acids including free amino acids, 3–8% lipids, 5–8% carbohydrates, 3% minerals, and 65–75%of moisture. However, the composition of BDH is also influenced by the region, season, and processing technique (Isidorov et al. 2016; Shoinbayeva et al. 2017). The presence of hormones, such as testosterone, oestradiol, progesterone, and prolactin, along with phenolic and flavonoid compounds with antioxidant properties, is likely primarily responsible for the positive effects of DBH on the male and female reproductive organs. Sex hormone levels change throughout the drone larval development, with more mature larvae showing increased testosterone and decreased progesterone and oestradiol (Sidor and Dzugan 2020; Ivgin Tunca and Arslan 2024). In addition, in some cultures, the drone larvae represent a valuable food source for humans. The biological and therapeutic properties of DBH have been demonstrated through a combination of laboratory assays and *in vivo* human evaluations (Guine et al. 2025). Based on the above findings, it appears that DBH may exhibit a range of medicinal and therapeutic properties, including immunomodulatory effects (Vasilenko et al. 2005). When using DBH, it is necessary – just as with other bee products – to be cautious and consider the possibility of an allergy to these products, since DBH may contain pollen, propolis, or nectar. If DBH is used in human or animal nutrition, it must be produced in accordance with standard safety and food-safety protocols, which include microbiological analyses and the determination of hazardous residues. Detailed information on the requirements for producing insect-based foods and feeds, including rearing procedures, is available from the International Platform of Insects for Food and Feed (https://ipiff.org).

The intestinal barrier comprises intestinal epithelial cells, the lamina propria, the mucus layer, and the microbiota. However, its integrity is also ensured by tight junctions (TJs), which consist of transmembrane proteins such as claudins and occludins, adherent junctions (AJs) and desmosomes. Adherent junctions, together with desmosomes, adhere directly to enterocytes, thereby ensuring their integrity, while TJs are located between the lateral parts of the enterocytes. TJs also include peripheral membrane proteins (zonula ocludens, regulatory proteins) (Buckley and Turner 2018). In this term, the lumican protein also actively participates in maintaining the intestinal barrier, which, in addition to activating the innate immune response, ensures mucosal lubrication in the individual sections of the small intestine (Smith and Melrose 2024).

It is generally assumed that an increased intestinal permeability contributes to the increasing incidence of gastrointestinal disorders. A pathologically altered intestinal barrier allows the subsequent translocation of bacteria into the organism’s internal environment, thereby provoking an inflammatory state (Kocot et al. 2022). Since there is insufficient information about the effects of DHB on the intestinal barrier and immune response, the aim of our study was to observe the impact of a dietary DHB supplementation in pigs on the relative gene expression of TJs proteins (occludin, claudin-1), as well as lumican and olfactomedin-4 in the ileum, and on the phagocytic activity and proportion of lymphocyte subpopulations in the peripheral blood.

## MATERIAL AND METHODS

### Production of the drone brood homogenate (DBH)

For the experimental production of drone larvae, six honeybee colonies were chosen. Each colony was maintained in a wooden B10 hive at an apiary in northern Slovakia (in the locality of Liptovský Hrádok – Fabriky), at 710 m above sea level. The production period covered May and June. Into the brood chamber, which consisted of 10 frames, two frames designated for comb construction and drone rearing were inserted in positions 3 and 8. The colonies were inspected every three days to monitor the queen’s oviposition and the development of the larvae. Once the drone larvae reached 6–7 days of age, the respective frames were removed. New drone frames were provided at 20-day intervals. The larvae were collected from these frames using the wash-up method: a gentle stream of water at 10–12 °C was applied to flush the larvae into a wooden sieve lined with a fibreglass mesh. Subsequently, the larvae were blotted on filter paper for 5–10 min to remove excess moisture, then placed in a freezer. The entire procedure, from frame removal to freezing, was completed within one hour, ensuring the preservation of the larval quality. The final processing involved freeze-drying in a laboratory lyophiliser (Heto PowerDry PL9000 –50 °C Shelf Freeze Dryer with an HSC 500 Plus Temperature Controller; Thermo Scientific, Waltham, MA, USA), operated at –49 °C and a vacuum of 0.086 hPa for 40–50 h, depending on the sample thickness. The lyophilised material was homogenised using an ultra-centrifugal mill with electronic control and cooling capacity (RETSCH ZM 200 Ultra Centrifugal Mill; Retsch GmbH, Haan, Germany) and subsequently stored at –18 °C until incorporation into the diet. The nutrient composition of DBH, including its amino acid, mineral, and vitamin content, was analysed and reported by Rajcakova et al. (2022). Bacterial contamination was excluded after culturing the DBH on nutrient media using the classical cultivation method.

### Animals

The experiment was carried out in the accredited menagerie (SK U 01019) of Laboratory of Physiology and Nutrition of Monogastric Animals (National Agricultural and Food Centre, Nitra, Slovakia). The experimental protocol (No. 4236/2022-220) was approved by the State Veterinary and Food Administration of the Slovak Republic. All the animals were handled and euthanised humanely, in compliance with the relevant commission’s guidelines and Directive 2010/63/EU of the European Parliament and Council on the protection of animals used for scientific purposes.

Eighteen 8-week-old hybrid Slovak White and Landrace pigs with an initial live weight of 19.5* ±* 2.7 kg were included in the 18-day trial. All the animals used in the experiment were clinically healthy, and their health status was monitored daily. The pigs were given a permanent ear tattoo and randomly divided into 3 groups (*n* = 6): DBH100, DBH200, and C (control). Each group contained 3 females and 3 males. The pig groups were separated by iron barriers and had free access to the starter feed (TEKRO Nitra, Ltd., Nitra, Slovakia) and water. Feed was composed of wheat (41.6%), barley (25%), extracted soybean meal (9%), fish meal (10%), dried whey (5%), monocalcium phosphate (0.65%), limestone (0.5%), salt (0.25%), and vitamin-mineral premix (5%). The nutrient composition of the diet corresponded to the commercial pig diet (Table 1) according to the feeding norms in Slovakia (TEKRO Nitra, Ltd., Nitra, Slovakia).

**Table 1:** Nutrient composition of the starter feed

Nutrient	Unit	Concentration
N-substances	g/kg	195
Fat	g/kg	55
Dietary fibre	g/kg	35
Lysine	g/kg	14
Methionine	g/kg	5.3
Calcium	g/kg	7.0
Phosphorus	g/kg	5.5
Sodium	g/kg	2.0
Copper	mg/kg	130
Zinc	mg/kg	2 500

The pigs in the DBH100 group were fed lyophilised DBH at a dose of 100 mg/kg of commercial feed, and the animals in the DBH200 group received a dose of 200 mg/kg of feed. The DBH-enriched feed was administered to the pigs for 18 days, twice daily – at 7:00 a.m. and 4:00 p.m. The pigs readily consumed the DBH-enriched feed, and no digestive problems were observed throughout the experiment. The animals in the control group received a standard commercial diet without DBH supplementation. On day 19, the pigs were weighed, peripheral blood was collected for the flow cytometric and haematological analyses, and they were subsequently euthanised in the experimental facility using an approved procedure. Subsequently, samples were collected for quantitative real-time polymerase chain reaction (PCR) (ileum).

### Homogenisation of the samples and isolation of the total RNA from the tissue samples

Samples of the caudal part of the ileum (20 mg weighted pieces) were immediately placed in an RNA later solution (Qiagen, Manchester, UK) and stored at –70 °C before the RNA purification and transcription, as described in detail in Karaffova et al. (2017).

### Quantitative real-time PCR method

The relative gene expression of selected markers involved in gut integrity (*olmf*4, lumican, occludin, claudin-1) was evaluated by quantitative real-time PCR using the SsoAdvanced^TM^ Universal SYBR Green Supermix kit (Bio-Rad Laboratories; Hercules, CA, USA) with specific primers (Table 2) on a LightCycler 480 II Instrument (Roche, Basel, Switzerland), following a predefined temperature programme. In addition, the mRNA relative expression of the reference gene encoding hypoxanthine-guanine phosphoribosyltransferase (HPRT) was determined using geNorm software based on expression stability. All the primer sets allowed DNA amplification efficiencies between 94% and 100%. The quantitative real-time (qRT)-PCR reaction was initiated by denaturation at 95 °C for 30 s, followed by 39 cycles of amplification: denaturation at 95 °C for 15 s, annealing at 60 °C for 30 s, and an elongation step at 72 °C for 2 minutes. A melting curve from 55 °C to 95 °C with readings at every 0.5 °C was recorded for each individual RT-qPCR plate. The analysis was performed after each run to ensure a single amplified product per reaction. Each real-time PCR reaction was performed in triplicate, and the mean values were used for further analysis.

**Table 2:** List of the primer sequences for the target genes

Primer	Sequence 5'→3'	References
*olfm*4 Fw	GTCAGCAAACCGGCTATTGT	Gonzalez et al. (2013)
*olfm*4 Rev	TGCCTTGGCCATAGGAAATA	
*lum* Fw	TCTGCTGGAGCTGGATCTCT	Paris-Oller et al. (2021)
*lum* Rev	CGCAAATGTTTGATCTTGGA	
*occl* Fw	CGGATTCTGTCTATGCTCGTTAT	Wang et al. (2020)
*occl* Rev	TAGCCCATACCACCTCCTATT	
*cldn-1* Fw	TGGTCAGGCTCTCTTCACTG	Liu et al. (2022)
*cldn*-1 Rev	TTGGATAGGGCCTTGGTGTT	
*hprt* Fw	AACCTTGCTTTCCTTGGTCA	Cinar et al. (2012)
*hprt* Rev	TCAAGGGCATAGCCTACCAC	

The obtained quantification cycle (*Cq*) values of the genes were normalised to the average *Cq* value of the reference gene, and the relative expression of each gene was calculated mathematically as 2^–Δ*Cq*^.

### Testing the activity of phagocytes in peripheral blood

A commercial flow cytometry test, Phagotest^®^ (Celonic, Munich, Germany), was used to measure phagocytic activity and absorption capacity. The principle of the test is to stimulate phagocytic activity using fluorescently labelled *E.* *coli*. The phagocytic activity expresses the percentage of the actively phagocytising cells and the absorption capacity expresses the average number of *E.* *coli* per phagocyte, expressed as the mean fluorescence intensity (MFI).

The phagocyte respiratory burst was assessed using a commercial Phagoburst test kit (Celonic, Munich, Germany), which stimulates phagocytosis with *E. coli.* Subsequently, in the phagocytes in which the respiratory burst occurs, the enzymes of the NADPH oxidase system split dehydrorhodamine 123 into fluorescent rhodamine 123. The test result is the percentage of cells in a respiratory burst and the level of the respiratory burst, expressed as the mean fluorescence intensity (MFI) per phagocyte. Peripheral heparinised blood was used for both tests, which were performed according to the manufacturer’s instructions. The tests were evaluated on a BD FACS Canto^TM^ flow cytometer (Becton, Dickinson and Company, San Jose, CA, USA) using BD FACS Diva^TM^ software.

### Lymphocyte phenotyping

The phenotyping of the selected lymphocyte subpopulations was performed in the peripheral blood taken from the supraorbital sinus of the pigs. A haematological analysis was performed using a BC-2008 VET automatic analyser (Mindray, Shenzhen, P.R. China). An aliquot of 50 μl of heparinised blood was mixed with fluorochrome-conjugated mouse anti-porcine monoclonal antibodies in two combinations: CD45/CD3e/CD21 and CD4/CD8a. For the analysis, 10 μl of anti-CD45-Alexa Fluor 647 (clone: K252.1E4; AbD Serotec, UK), 4 μl of anti-CD3e-FITC (clone: BB23-8E6; BD Biosciences, USA), and 2 μl of anti-CD21-RPE (clone: LT-21; ThermoFisher Scientific, USA) were used. In the second combination, 4 μl of anti-CD4-FITC (clone: MIL 17; AbD Serotec, UK). Blood with antibodies was incubated for 20 min in the dark at 25 °C and then erythrocytes were removed using 1 ml of a lysis solution (BD FACS lysing solution, USA) for 20 min again in the dark at 25 °C. The samples were centrifuged at 300 × *g* for 5 minutes. The resulting cell pellets were washed twice with 1 ml of phosphate-buffered saline (PBS; MP Biomedicals, France), where each wash was followed by centrifugation under the same conditions. After the final wash, the cells were resuspended in 200 μl of PBS for flow cytometry analysis. The flow cytometric analysis was performed using the above-described cytometer, acquiring data from 10 000 labelled lymphocytes. The results were analysed using dot-plot histograms and expressed as the relative percentages of the different lymphocyte subpopulations. The CD45-positive cells within the lymphocyte gate were greater than 99%.

### Statistical analysis

The statistical analysis of the data was performed using GraphPad Prism v9 software. Data normality was tested with the Shapiro–Wilk test. Since all datasets showed normal distributions, a one-way analysis of variance (ANOVA) followed by Tukey’s post hoc multiple-comparison test was used for data analysis. The results are expressed as averages and standard deviations. Differences between the group values were considered statistically significant at *P < 0.05; **P < 0.01; ***P < 0.001.

## RESULTS

**Figure 1:**
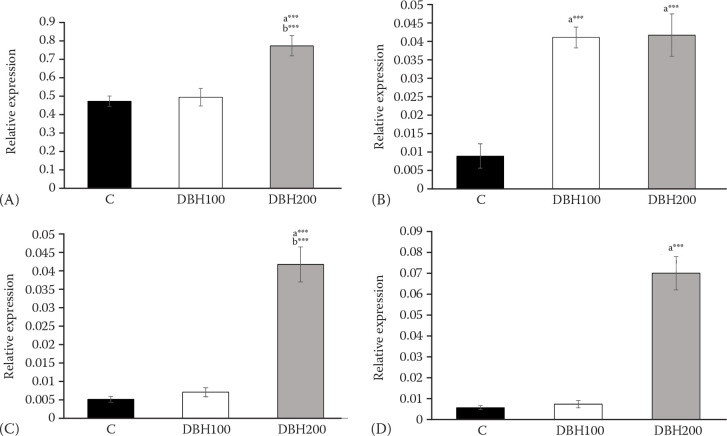
The influence of the administration of DBH to the pigs (*n* = 6) on the relative expression of genes for: (A) *olfm4*; (B) *lum*; (C) *occl*; (D) *cldn-1* in the ileum ^a^Significantly different from the control ©; ^b^Significantly different from the group DHB100 ***P < 0.001

The final body weights of the pigs did not differ significantly; however, we observed a tendency toward higher weights in the DBH groups (DBH100: 37.8 ± 4.2 kg; DBH200: 38.4 ± 3.8 kg) compared to the control group (36.4 ± 1.1 kg). The relative gene expression for *olfm4* was markedly up-regulated in the DBH200 group compared to the other groups (*P* < 0.000 1) (Figure 1A). The relative gene expression for lumican was up-regulated in both the experimental groups in comparison with the control (*P* < 0.000 1) (Figure 1B). On the other hand, the occludin gene expression was the highest in the DBH200 group compared with the DBH100 group and control (*P* < 0.000 1) (Figure 1C). The same tendency was noted for the *cldn-1* gene expression, which was significantly up-regulated in the DBH200 group compared to the other groups (*P* < 0.000 1) (Figure 1D).

**Figure 2:**
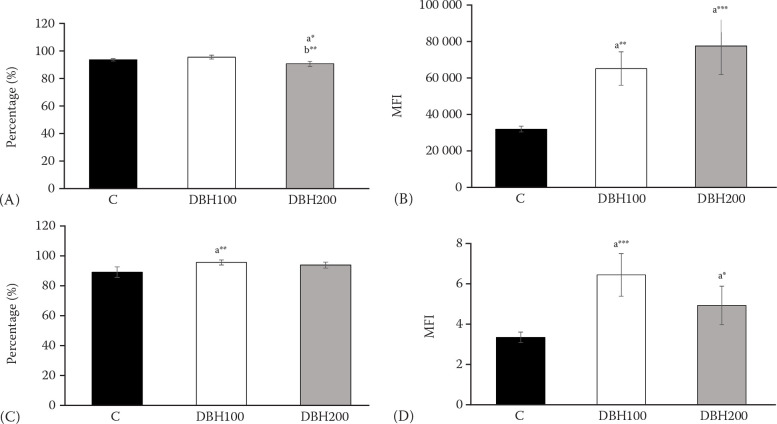
The influence of DBH on the activity of phagocytes in the pig blood was evaluated as: (A) The percentage of active phagocytes (phagocytic activity); (B) the engulfing capacity of phagocytes (expressed as mean fluorescence intensity – MFI); (C) the oxidative burst; and (D) the level of oxidative burst ^a^Significantly different from the control ©; ^b^Significantly different from the group DHB100 *P < 0.05; **P < 0.01; ***P < 0.001

The assessment of the impact of the drone brood homogenate on the phagocyte activity in the porcine blood revealed only a moderate effect on the percentage of active phagocytes. In the DBH200 group, phagocytic activity decreased significantly compared to both the control and DBH100 groups (Figure 2A). In contrast, the engulfing capacity of phagocytes was significantly higher in both experimental groups than in the control, with higher values in the DBH200 group than in the DBH100 group, although this difference was not statistically significant (Figure 2B). Regarding the oxidative burst activity of phagocytes, the percentage of active phagocytes was significantly higher in the DBH100 group than in the control, whereas in the DBH200 group, the increase was not statistically significant (Figure 2C). The oxidative burst index was significantly elevated in both experimental groups compared to the control (Figure 2D).

**Figure 3:**
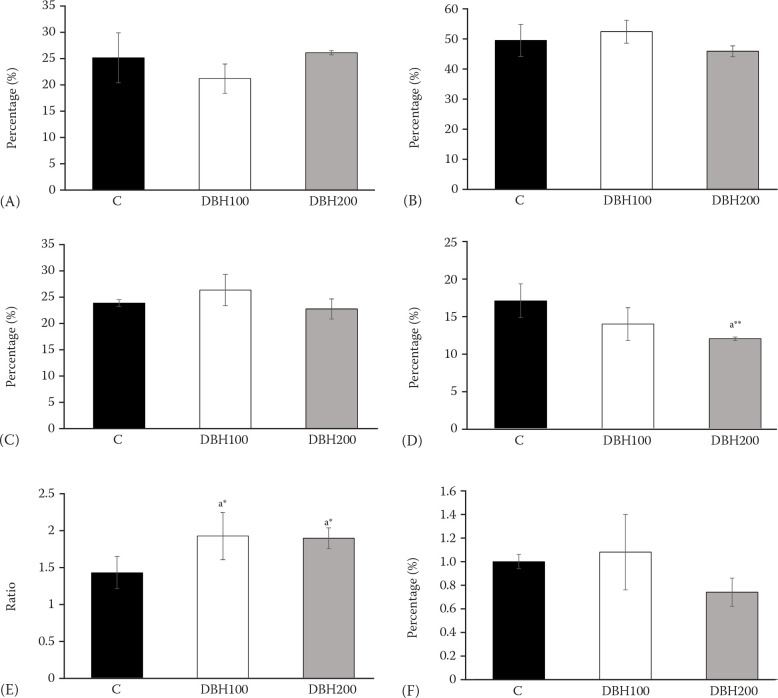
Percentage of (A) CD21^+^; (B) CD3^+^; (C) CD4^+^CD8^–^; (D) CD4^–^CD8^+^; (E) Ratio CD4^+^ : CD8^+^; (F) CD4^+^CD8^+^ lymphocytes in the blood of the pigs fed DBH (*n* = 6) ^a^Significantly different from the control ©; ^b^Significantly different from the group DHB100; ^c^Significantly different from the group P *P < 0.05; **P < 0.01

The application of DBH had no effect on the haematological parameters in the pigs’ peripheral blood (results not shown). The CD21^+^ and CD3^+^ lymphocyte subpopulations, representing B and T cells, respectively, were only minimally affected by the DBH administration. In the DBH100 group, a decreasing trend in the proportion of B lymphocytes and a corresponding increase in T lymphocytes were observed (Figure 3A,B). The proportions of individual lymphocyte subpopulations were also converted to absolute counts. While the number of B lymphocytes (CD21^+^) differed minimally between the groups, a tendency toward increased T lymphocytes (CD3^+^) was observed in the DBH100 group compared with both the DBH200 and control groups, consistent with the percentage representation of T lymphocytes. Regarding T lymphocyte subpopulations, a tendency toward a higher proportion of Th lymphocytes (CD4^+^CD8^–^) was observed in the DBH100 group compared with both the control and DBH200 groups (Figure 3C). Conversely, the percentage of Tc lymphocytes (CD4^–^CD8^+^) was significantly lower in the DBH200 group compared to the control, while a decreasing trend was observed in the DBH100 group (Figure 3D). The CD4^+^ : CD8^+^ ratio, an indicator of immune stimulation, was significantly increased in both DBH-treated groups compared with the control (Figure 3E). The percentage of double-positive lymphocytes was not affected by the DBH administration (Figure 3F).

## DISCUSSION

The use of natural substances, including bee products, is gaining in importance. In this context, bee products are well known for their pronounced antibacterial and antioxidant effects. The main antioxidant components include polyphenolic compounds, vitamins C and E, enzymes, and other biologically active substances (Gheldof et al. 2002). Comparative studies have demonstrated that propolis shows the highest antioxidant activity, followed by bee pollen and drone brood homogenate. At the same time, it has been shown that drone brood homogenate contains the highest amount of polyphenolic compounds among the analysed bee products (Sidor and Dzugan 2020). Due to its rich nutritional composition, especially its hormone content, Apilarnil is primarily used in pigs to improve reproductive health; it enhances fertility in gilts, increases semen production in boars, and acts as a gonad protector. In addition, its anabolic effect improves the production parameters in pig fattening (Bolatovna et al. 2015). In addition to the effects mentioned above, it is essential, with regard to the prospective practical use of DBM in animal production, to investigate its impact on the permeability and preservation of intestinal integrity.

It is well established that tight junction (TJ) proteins help maintain intestinal barrier function. Epithelial TJs consist of multiple junctional components, including claudins, occludins, and zonula occluden proteins, which regulate the paracellular passage of macromolecules, ions, and water between adjacent cells. Occludin is a major transmembrane constituent of TJs and, together with claudin-1, plays a crucial role in maintaining intestinal permeability and overall barrier integrity (Neurath et al. 2025).

Within this framework, the glycoprotein OLFM4 and the proteoglycan lumican play significant roles, particularly in innate immunity against bacterial pathogens, as well as in inflammatory diseases of the gastrointestinal tract and certain cancers, thereby actively contributing to the maintenance and strengthening of gut integrity (Karamanou et al. 2018). Recent studies have also demonstrated that both proteins are essential for preserving the mucosal integrity, particularly during wound healing (Smith and Melrose 2024; Liu and Rodgers 2025). In this regard, our study demonstrated that supplementing pig feed with 200 mg/kg of DBH significantly increased the expression of all selected genes compared with the control group. A positive effect on gut health is also supported by the slightly higher body weights of the pigs after 18 days of DBH administration compared with the control group, although the differences were not statistically significant. We hypothesise that this could also be due to the relatively high content of vitamin A in DBH, which is essential for maintaining vision and immune function, supporting growth and development, preserving epithelial cell integrity, and ensuring reproduction (Silici 2023). It could also be attributable to the presence of chitin, a precursor of chitosan, since chitin-mediated barrier immunity is considered to be an ancient defence mechanism, the loss of which may impair epithelial protection and host resistance against microbial invasion (Nakashima et al. 2018). Moreover, it turns out that chitin and its derivatives can serve as potential prebiotics for the intestinal microbiota, thereby contributing to and strengthening the intestinal barrier’s protection against pathogens (Kipkoech et al. 2021).

Similarly, Yassien et al. (2024) reported that administering another product, bee drone milk, resulted in beneficial regulation of oxidative stress, apoptosis, and inflammation in rat testes.

Furthermore, although a number of studies have characterised the morphological composition, properties, and potential applications of bee drone brood, investigations into its specific interactions with tight junction proteins and costimulatory molecules that maintain intestinal barrier integrity are still lacking.

Although several publications state that DBH has immunostimulatory or immunomodulatory effects, the supporting evidence is mostly lacking. Vasilenko et al. (2005) tested the effect of lyophilised drone larvae on selected parameters of the innate immunity in rats with chemically induced hepatitis. They noted a significant increase in the phagocyte activity and beta-lysine levels, as well as a decrease, (i.e., normalisation), of the lysozyme levels compared to untreated control rats with hepatitis.

Inandiklioglu et al. (2021) confirmed the anti-inflammatory effect of Apilarnil, evidenced by reduced gene expression of pro-inflammatory cytokines (IL-6, IL-1β, TNF-α) and TLR-4, as well as nuclear factor κB, in kidney tissue. Arican et al. (2025) investigated the effect of topical DBH application on wound healing in rats and evaluated lymphocyte, polymorphonuclear leukocyte, and fibroblast infiltration. In this study, no effect on these parameters was observed; however, the DBH application increased the scar density and wound contraction. In 2024, Ivgin Tunca and Arslan (2024) published a comprehensive review of Aapilarnil, including its effects on human and animal health. However, none of the published studies investigated the effect of DHB on the lymphocyte subpopulations.

In our study, the engulfing capacity of phagocytes was significantly increased after DHB administration, whereas the percentage of active phagocytes was only weakly affected. Moreover, after administering a higher dose of DHB (200 mg), we observed a significant decrease in the percentage of active phagocytes. Regarding the oxidative burst of phagocytes, DHB significantly increased the oxidative burst index compared to the control; however, once again, the percentage of phagocytes exhibiting oxidative burst was only weakly affected. Regarding the effect of DBH on lymphocyte subpopulations, we did not observe a significant effect on total T lymphocytes (CD3^+^) or B lymphocytes (CD21^+^). However, in the DBH100 group, a slight tendency toward an increase in CD3^+^ lymphocytes was recorded compared to the control, which was subsequently reflected in a non-significant increase in the proportion of the T helper lymphocyte subpopulation (CD4^+^CD8^−^). In contrast, the percentage of cytotoxic T lymphocytes (CD4^−^CD8^+^) was lower in both experimental groups than in the control, with a significant difference in the DBH200 group and a non-significant difference in the DBH100 group. As a result of these shifts, both experimental groups showed a statistically significantly higher CD4^+ ^: CD8^+^ lymphocyte ratio, an indicator of immunostimulation.

A dietary supplementation of drone brood homogenate (DBH) in pigs enhanced the expression of intestinal barrier–related genes, including occludin, claudin-1, lumican, and *olfm4*, indicating improved mucosal integrity. DBH also increased the phagocytic capacity and oxidative burst activity of the peripheral blood phagocytes, although the proportion of actively phagocytising cells was minimally affected. While the total T and B lymphocyte populations remained largely unchanged, both DBH-treated groups exhibited a significantly higher CD4^+^ : CD8^+^ ratio, suggesting immunostimulatory effects. Overall, DBH demonstrates potential as a natural feed additive to support the intestinal barrier function and modulate key aspects of innate and adaptive immunity in pigs.
